# Reduced Field-of-view Diffusion-Weighted Magnetic Resonance Imaging for Detecting Early Gastric Cancer: A Pilot Study Comparing Diagnostic Performance with MDCT and fFOV DWI

**DOI:** 10.2174/0115734056390767250917221319

**Published:** 2025-09-24

**Authors:** Guodong Song, Guangbin Wang, Leping Li, Liang Shang, Shuai Duan, Zhenzhen Wang, Yubo Liu

**Affiliations:** 1 Department of Radiology, Shandong Provincial Hospital Affiliated to Shandong First Medical University, Jinan, Shandong, China; 2 Department of Gastrointestinal Surgery, Shandong Provincial Hospital Affiliated to Shandong First Medical University, Jinan, Shandong, China; 3 Department of Radiology, People’s hospital of Rongcheng, Rongcheng City, Shandong, China; 4 Department of Radiology, Shandong Provincial Hospital, Cheeloo College of Medicine, Shandong University, Jinan, Shandong, China

**Keywords:** Diffusion-weighted imaging, Early gastric cancer, Full field-of-view, Reduced field-of-view, Magnetic resonance imaging, Computed tomography

## Abstract

**Introduction:**

Early detection of gastric cancer remains challenging for many of the current imaging techniques. Recent advancements in reduced field-of-view (rFOV) diffusion-weighted imaging (DWI) have shown promise in improving the visualization of small anatomical structures. This study aimed to evaluate and compare the diagnostic performance of rFOV DWI with multi-detector computed tomography (MDCT) and conventional full field of view (fFOV) DWI for detecting early gastric cancer (EGC).

**Methods:**

This retrospective study included 43 patients with pathologically confirmed EGC. All participants underwent pre-treatment imaging, including CT scans and MRI with a prototype rFOV DWI and conventional fFOV DWI at 3 Tesla. Quantitative (signal-to-noise ratio [SNR], contrast-to-noise ratio [CNR]) and qualitative (subjective image quality) assessments were performed. Diagnostic performance was evaluated using receiver operating characteristic (ROC) curves and area-under-the-curve (AUC) analysis.

**Results:**

rFOV DWI demonstrated significantly higher SNR and CNR compared with fFOV DWI (*P* < 0.05). Subjective image quality scores were also superior for rFOV DWI (*P* < 0.05). In lesion detection, rFOV DWI showed higher sensitivity (0.705) than CT (0.636) and fFOV DWI (0.523). ROC analysis revealed that rFOV DWI had a higher AUC (0.829, 95% CI [0.764, 0.882]) than fFOV DWI (0.734, 95% CI [0.661, 0.798], *P* = 0.02) and a modest improvement over CT (0.799, 95% CI [0.731, 0.856], *P* = 0.51).

**Discussion:**

The findings suggest that rFOV DWI provides superior image quality and diagnostic accuracy for EGC detection compared with conventional fFOV DWI. While it showed a trend toward better performance than CT, further studies with larger cohorts are needed to validate these results.

**Conclusion:**

rFOV DWI offers improved image quality and diagnostic performance for early gastric cancer detection compared with fFOV DWI, with a potential advantage over CT. This technique may enhance early diagnosis and clinical decision-making in gastric cancer management.

## 1 INTRODUCTION

Gastric cancer is the fifth most common cancer and the fourth leading cause of cancer-related mortality worldwide [1]. Most cases are diagnosed at advanced stages, with relatively worse outcomes [2]. Early gastric cancer (EGC) is a unique form of gastric carcinoma characterized by tumor invasion limited to the mucosa or submucosa, regardless of lymph node metastasis [3]. This condition is classified as a T1 stage tumor according to the 8th edition of the American Joint Committee on Cancer. Early detection of EGC is clinically critical, as patients with T1 tumors can often be treated with endoscopic resection or minimally invasive surgery, potentially avoiding the need for neoadjuvant therapy. Studies have shown that EGC has an excellent prognosis, with five-year survival rates exceeding 90% following curative resection [4].

Multi-detector computed tomography (MDCT) is a routine imaging method used in the preoperative evaluation of gastric cancer; however, the results are heterogeneous, especially in the early stages of the tumor. Both CT and magnetic resonance imaging (MRI) are known to have a limited role in detecting EGC [5, 6]. Early detection of gastric cancer is challenging for many of the current imaging techniques.

Diffusion-weighted imaging (DWI) is a functional MRI technique that evaluates tumors without using contrast material. The apparent diffusion coefficient (ADC) value derived from DWI is a promising imaging biomarker for tumor assessment. Studies have demonstrated that DWI and ADC values correlate with various histopathological features, treatment responses, tumor aggressiveness, and prognosis in gastric cancer [7-9].

However, conventional single-shot echo-planar imaging (EPI) DWI sequences face several challenges, including peristaltic motion, respiratory artifacts, and lower spatial resolution, resulting in relatively poor image quality [10]. These limitations restrict their routine use for imaging gastric tumors, particularly EGCs. Therefore, a DWI sequence that provides high resolution and excellent image quality may be essential for improving EGC detection.

Recently, reduced field-of-view (rFOV) DWI has emerged as a promising approach to improve image quality for small anatomical structures in various organs [11-15]. rFOV DWI uses two-dimensional spatially selective excitation pulses to narrow the phase-encoding FOV, reducing the required k-space lines. Compared with conventional full-FOV (fFOV) DWI, rFOV DWI combines EPI with a parallel RF pulse sequence to enhance signals from the region of interest while improving anatomical detail. This technique significantly reduces distortion, blurring, motion artifacts, and folding artifacts, while enhancing spatial resolution and image fidelity. Additionally, it enables faster scanning, making rFOV DWI a more efficient and high-quality method with fewer artifacts and geometric distortions than full-FOV DWI [11, 16, 17]. These advantages may be particularly beneficial for detecting small lesions such as early-stage gastric tumors.

Therefore, the purpose of this study was to compare the image quality between rFOV DWI and fFOV DWI and to investigate the potential role of rFOV DWI in detecting EGC.

## 2 MATERIALS AND METHODS

### 2.1 Patients

This study was approved by our institutional review board (No. 2024-162) and conducted in accordance with the latest version of the Declaration of Helsinki. Informed consent was waived because of the retrospective nature of the study. From January 2021 to December 2024, 148 patients who underwent both CT and MRI examinations (including rFOV DWI and fFOV DWI) were reviewed retrospectively. The inclusion criteria for the study were: 1) a confirmed diagnosis of EGC through pathology; 2) no treatment or biopsy conducted within two weeks before the CT and MRI scans; and 3) the completion of both rFOV DWI and fFOV DWI sequences during the MRI examination. The exclusion criteria included: 1) a confirmed diagnosis of non-EGC through pathology; and 2) incomplete medical records.

### 2.2 CT and MR Examination

After fasting for 6-8 hours, patients were instructed to ingest 800-1000 mL of water to distend the stomach before the examination.

MDCT was performed using a dual-source CT scanner (SOMATOM Definition Force; Siemens Healthcare, Erlangen, Germany). The parameters for the CT scanner were set as follows: tube voltage of 100 or 120 kVp, tube current ranging from 250 to 400 mA, a matrix size of 512 × 512, and a rotation time of 0.25–0.60 seconds. Patients received intravenous contrast (Omnipaque, GE Healthcare) based on their body weight, at a dosage of 1–2 mL/kg, with an infusion rate of 3 mL/s. The arterial phase was initiated using bolus tracking, followed by the portal phase and delayed phase, which began 35 and 120 seconds after the arterial phase scan, respectively. The examination was conducted in a spiral format, with a slice thickness of 5 mm and a reconstruction slice thickness of 1.25 mm.

MR examinations were performed using a 3 T MR imaging system (MAGNETOM Prisma, Siemens Healthcare, Erlangen, Germany) with an 18-channel body coil. For each patient, T2-weighted imaging (T2WI), rFOV DWI, and fFOV DWI sequences were acquired before treatment. A BLADE TSE T2WI sequence in the axial plane (TR: 2890 ms, TE: 94 ms, slice thickness: 5 mm, FOV: 380 mm× 380 mm, acquisition time: 1 min 50 s) was obtained before the DWI sequence. Conventional fFOV DWI using a transversal EPI sequence (TR: 4000ms, TE: 47ms, b-value: 50, 800 s/mm^2^, slice thickness: 5mm, FOV: 298 mm × 399 mm, Acquisition Matrix: 134 × 100; acquisition time: 1 min 59 s) was also acquired. Additionally, a zoomed DWI using a prototypical reduced field of view EPI sequence with a tilted excitation plane [18] (TR: 2500ms, TE: 68ms, b-value: 50, 800, 1000, 1500 s/mm^2^, slice thickness: 5 mm, FOV: 118 mm × 259 mm, Acquisition Matrix: 110 × 50, acquisition time: 2 min 34 s) was performed. This tilted rFOV-DWI eliminates phase-encoding aliasing artifacts by slightly rotating the spatially selective 2D RF excitation plane, which displaces side excitations outside the image plane [18].

### 2.3 Qualitative Image Analysis

All the CT, rFOV, and fFOV DWI images were transferred to a picture archiving and communication system (PACS) for assessment by two independent abdominal radiologists, each with over six years of experience. Both reviewers were blinded to the clinical data and pathological results, and the optimal window setting was adjusted for each case. Consistent with prior studies [19], the stomach was segmented into four regions: the cardia (or fundus), body, antrum, and pylorus. Furthermore, following the 8th edition of the American Joint Committee on Cancer (AJCC) Staging Criteria, the two radiologists independently assigned a final diagnosis regarding the presence of EGC for each of the three imaging sequences. This evaluation was conducted across a total of 132 gastric segments and utilized a 5-point Likert scale: 1 indicating definitely absent, 2 indicating probably absent, 3 indicating possibly present, 4 indicating probably present, and 5 indicating definitely present.

For the evaluation of the two DWI sequences, the reviewers determined overall image quality (1= poor, 2=fair, 3=good, 4=excellent), anatomic detail display (1=poor, 2=fair, 3=good, 4=excellent), distortion (1=severe, 2=moderate, 3=slight, 4=absent), lesion conspicuity (1=poor, considered unidentifiable, 2=fair, lesions with slight signal difference, 3=good, lesions identifiable, 4=excellent, clearly identifiable), and artifacts (1=serious, 2=moderate, 3=slight, 4=absent). In addition, the quality of perigastric lymph node detection was also evaluated (1=poor, 2=fair, 3=good, 4=excellent). The reviewers first assessed T2-weighted images using fFOV DWI, followed by rFOV DWI, and finally CT images. They used the same criteria at a two-week interval to minimize recall bias.

### 2.4 Quantitative Image Analysis

Regions of interest (ROIs) were drawn using only those lesions rated 3 or higher by the reviewers. These ROIs were manually placed on axial DWI images to cover the maximum dimension of tumors and then copied to the ADC map. The placement of ROIs was achieved by delineating the margin of the entire tumor.

As previously described [20], the signal-to-noise ratio (SNR) of rFOV and fFOV DWI was calculated as the mean signal intensity of the ROI divided by the standard deviation (SD) of the background noise. The noise level was measured in the artifact-free background region [21]. The contrast-to-noise ratio (CNR) of rFOV and fFOV DWI was calculated based on the following eq. (**1**):

**Table:** 

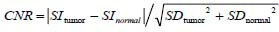	(1)

where SI_tumor_ and SI_normal_ represent the mean signal intensity of the tumor and normal perigastric fat tissue, respectively. The ADC values were calculated using a monoexponential function with low (b = 50) and high (b = 800) b values. All measurements were performed twice on the same slices with an interval of two weeks, and the average values were recorded.

### 2.5 Statistical Analysis

All statistical analyses were carried out on SPSS version 28.0 (IBM, Armonk, NY). The interobserver agreement on qualitative analysis was assessed using kappa statistics (0.00 − 0.20, poor repeatability; 0.21 − 0.40, fair; 0.41 − 0.60, moderate; 0.61 − 0.80, good; > 0.81, excellent reliability). The qualitative image scores for the rFOV DWI and fFOV DWI sequences were assessed using the Wilcoxon signed-rank test. Mean values for ADC, SNR, and CNR between the two groups were assessed using either Student’s t-test or the Wilcoxon signed-rank test, depending on the results of the normality test, with effect sizes calculated using Cohen’s d. Data normality was checked using the Shapiro-Wilk test. The diagnostic performance of the lesions was assessed using a receiver operating characteristic (ROC) curve. The area under the curve (AUC) was calculated, and differences were compared using the Z test. Similar to the selection of ROIs, sensitivity, specificity, and overall accuracy were calculated using only those lesions assigned a rating of 3 or higher. The ROC curves were created by MedCalc Statistical Software v. 20.022 (MedCalc Software, Ostend, Belgium). All data are reported as mean ± SD.

## 3 RESULTS

The flow chart of patient selection is shown in Fig. (**1**). A total of forty-three patients with 44 lesions were ultimately enrolled and analyzed in this study, comprising 17 females and 26 males. The baseline characteristics are shown in Table **1**. EGCs were diagnosed through gastrectomy or endoscopic resection. Among these lesions, 19 EGCs invaded the lamina propria or muscularis mucosae (T1a), while 25 tumors were confined to the submucosa (T1b). Regarding lesion location, 24 lesions were found in the gastric antrum, 6 in the gastric body, 12 in the cardia or fundus, and 2 in the pylorus.

### 3.1 Imaging Analysis

The interobserver agreement between readers was good to excellent for the two sequences in the qualitative analysis (rFOV-DWI: 0.673-0.890, fFOV-DWI: 0.658-0.909).

As shown in Table **2**, the SNR and CNR values were significantly higher in the rFOV DWI group than in the fFOV DWI group (*P* < 0.05). Furthermore, all qualitative scores related to image quality were higher in the rFOV group than in the fFOV DWI group (*P* < 0.05), encompassing overall image quality, lesion conspicuity, anatomic details, and artifacts. Representative examples are shown in Figs. (**2** and **3**). Notably, the detection of perigastric lymph nodes did not reveal any significant differences between the two groups (*P* > 0.05).

### 3.2 Detection of EGCs

Table **3** and Fig. (**4**) present a comparison of the diagnostic accuracy of CT, rFOV, and fFOV DWI sequences in detecting EGCs. Compared with both CT and fFOV DWI, rFOV DWI demonstrated superior performance, with higher sensitivity (0.705 *vs.* 0.636 and 0.523, respectively) and accuracy (0.890 *vs.* 0.878 and 0.837, respectively). The AUC of rFOV DWI (0.829, 95% CI: 0.764-0.882) was significantly higher than that of fFOV DWI (0.734, 95% CI: 0.661-0.798, *P* = 0.02), and it also outperformed CT (AUC: 0.799, 95% CI: 0.731-0.856), although the difference with CT was not statistically significant (*P* = 0.51). Additionally, while CT showed a better AUC than fFOV DWI (0.799 compared with 0.734), this difference did not reach statistical significance (*P* = 0.09).

A total of 13 lesions were missed by rFOV DWI, including 8 pT1a (mucosal) and 5 pT1b (submucosal) tumors, whereas fFOV DWI failed to detect 21 lesions, 13 of which overlapped with those missed by rFOV DWI. Among the 21 undetected lesions in the fFOV DWI group, 11 (52.4%) were mucosal (pT1a) and 10 (47.6%) showed submucosal invasion (pT1b). Notably, 69.2% (9/13) of lesions undetected by rFOV DWI were located in the gastric antrum, underscoring a regional diagnostic limitation. Additionally, missed lesions on rFOV DWI were significantly smaller than the detected ones (*P*= 0.004, Table **S1**).

### 3.3 ADC value comparison

Among the lesions accurately detected both on rFOV DWI and fFOV DWI, the ADC values of the rFOV DWI group were significantly lower than those of the fFOV DWI group (1.16±0.19×10^-3^ mm^2^/s *vs.* 1.33±0.21×10^-3^ mm^2^/s, respectively; *P* < 0.001)(Table 2). In contrast, as shown in Table **4**, there were no significant differences in ADC values between T1a and T1b-stage tumors for either the rFOV or fFOV groups (all *P* > 0.05).

## 4 DISCUSSION

In this retrospective study, we used a reduced FOV DWI sequence and compared this protocol with a conventional full FOV DWI sequence and MDCT in 43 patients with EGCs. Our results demonstrate that rFOV DWI provides superior image quality, as well as higher CNR and SNR in the imaging of EGC compared with fFOV DWI. Additionally, rFOV DWI demonstrated significantly superior tumor detection capability compared with fFOV DWI. The diagnostic performance of rFOV DWI is comparable to that of CT. Incorporating rFOV DWI into routine protocols may enhance the detection of EGCs.

Imaging the gastrointestinal tract with MRI, including DWI, is challenging due to the long acquisition time, susceptibility to intraluminal gastric gas, and motion artifacts, all of which can compromise image quality [22]. Conventional MR imaging of the stomach has shown low accuracy in detecting early gastric cancers [23]. For early gastric cancer, DWI is more sensitive than T2-weighted protocols, and adding DWI to conventional MR sequences improves the accuracy of T-staging [24, 25]. However, traditional single-shot EPI DWI remains a challenging technique because of its inherently limited spatial resolution, especially in abdominal imaging [17].

The advancement of the rFOV technique allows for potential application in various small organs or lesions, offering relatively higher resolution and improved image quality compared with fFOV DWI [13, 14]. Although previous studies have shown mixed agreement, most suggest a promising prospect of rFOV DWI in detecting various tumors [16]. Our study confirmed the feasibility of using rFOV DWI to detect EGCs, with improved detectability due to enhanced spatial resolution, which allows for better visualization of EGCs compared with fFOV DWI. This was similarly noted in other tumors, such as insulinomas [14] and bladder cancer [26]. Additionally, both DWI sequences exhibited good to excellent interobserver agreement in qualitative analyses.

Early detection remains paramount for EGC given its rising incidence and excellent prognosis with proper treatment. Our findings suggest that rFOV DWI can assist in detecting EGCs noninvasively, without the risk of ionizing radiation. This aligns with a recent study [27], which demonstrated that multiparametric MRI, including zoomed DWI, exhibited higher accuracy in identifying pT1 tumors compared to dual-energy CT. While rFOV DWI showed superior detection rates than fFOV DWI (13 *vs.* 21 missed lesions), it still has limitations in detecting small (particularly pT1a) and antral tumors (69.2%). A previous study [28] has highlighted regional variations in image quality, with optimal clarity at the esophagogastric junction due to its stable central position when distended, while the antrum is more susceptible to motion artifacts. These technical constraints, in addition to minimal diffusion changes in early-stage lesions, may explain rFOV DWI’s reduced sensitivity for small and antral tumors. Consequently, rFOV DWI should be used in conjunction with endoscopy, especially for detecting antral and early-stage gastric cancers.

In our study, we observed a significant difference in ADC values between rFOV DWI and fFOV DWI. Specifically, the rFOV DWI sequence showed lower ADC values relative to the fFOV DWI sequence, which is consistent with findings from previous studies [26, 28, 29]. Several researchers [26, 29] have reported that the ADC values obtained with rFOV DWI in muscle-invasive bladder cancer and rectal cancer were significantly lower than those derived from fFOV DWI. Similar results have been documented in gastric cancer [28] and head and neck tumors [30]. The reduction in ADC values with rFOV DWI is likely due to decreased partial volume effects between the tumor and adjacent normal tissue, resulting in more accurate values that better represent the true ADC of the tissues [28, 31-33]. The lower ADC values obtained with rFOV DWI enhance tumor-to-normal tissue contrast, which may improve the detection of challenging EGC subtypes, such as early signet-ring cell carcinomas that often evade detection with conventional imaging. Furthermore, the ability of this technique to resolve intratumoral ADC heterogeneity may aid in preoperative risk stratification, potentially guiding decisions between endoscopic resection and surgical intervention for borderline T1/T2 lesions. Conversely, some studies focused on cervical cancer [12, 34] and the gallbladder [35] found no significant differences in mean ADC values between rFOV DWI and fFOV DWI. The ADC values in vivo may be influenced by various technical factors, including magnetic field strength, choice of b values, echo time, site, and size of sampling methods. The discrepancies observed across different studies suggest that quantitative ADC values remain a subject of contention, necessitating further research to clarify changes and the repeatability of ADC values under various conditions.

To date, CT remains the preferred imaging modality for gastric cancer assessment due to its non-invasive capability to evaluate local tumor extension, nodal disease, and metastases. In contrast, MRI provides superior soft tissue contrast and multiple sequences without radiation risks. Several studies [23, 36] have shown that MRI's accuracy in T-stage assessment surpasses that of CT, particularly in detecting T1 lesions [37]. This suggests that MRI may be more effective in identifying EGC. In comparison to CT or conventional morphological MRI, DWI is more sensitive in detecting subtle or early tumor changes, and a meta-analysis has confirmed that DWI is more effective for T staging [38]. Soydan *et al.* [39] reported a sensitivity of 92.1%, specificity of 75%, and accuracy of 89.1% for ≤T2 *vs.* ≥T3 lesions using diffusion-weighted MRI. Our results also suggest that rFOV DWI exhibits higher sensitivity and AUC than CT in detecting EGC, although the difference is not statistically significant. This indicates that rFOV DWI could be a powerful sequence for EGC assessment. Additionally, some studies have emphasized the enhanced value of combining DWI and conventional MRI for preoperative TNM staging [24, 25]. Therefore, we believe MR imaging with rFOV DWI is superior in gastric cancer assessment and a valid alternative to CT in clinical settings.

Our study still has some limitations. First, selection bias may be present due to the retrospective, single-center design and the relatively small sample size. Additionally, the performance of rFOV DWI might be overestimated since only confirmed EGC cases were included in our study cohort. To confirm and validate our findings, larger prospective studies involving both healthy volunteers and EGC patients are necessary. Second, due to the reduced FOV, potential lesions outside the FOV might be missed, such as metastases in other organs or lymph node enlargement beyond the FOV. Therefore, a full FOV scan is still needed in routine protocols for comprehensive evaluation. Furthermore, although MRI is not routinely used for clinical detection of EGC, it could be a valuable tool for evaluating gastric tumors due to its high resolution and superior image quality, particularly for patients with contraindications to intravenous CT contrast agents. We propose that rFOV DWI can be an effective triage tool for symptomatic patients in endoscopic screening programs, ensuring appropriate prioritization of referrals. In biopsy-confirmed cases, its superior spatial resolution may aid in accurate lesion localization and depth assessment. Additionally, rFOV DWI can offer a radiation-free alternative to CT for post-treatment monitoring and detecting local recurrence. Further studies are necessary to explore these potential advantages.

## CONCLUSION

In summary, our study showed that applying rFOV DWI in EGC patients is promising, with high resolution and better image quality. Moreover, our data suggest that rFOV DWI performs better in detecting EGC, with higher diagnostic confidence than that of fFOV DWI and CT. Thus, rFOV DWI may serve as a potential tool for improving the diagnostic accuracy of early-stage gastric cancer.

## Figures and Tables

**Fig. (1):**
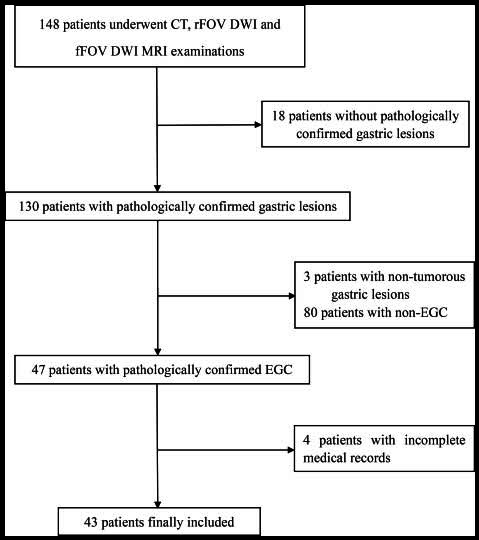
The flowchart for this study. EGC, early gastric cancer, rFOV DWI, reduced field-of-view diffusion-weighted imaging, fFOV DWI, full field-of-view diffusion-weighted imaging.

**Fig. (2):**
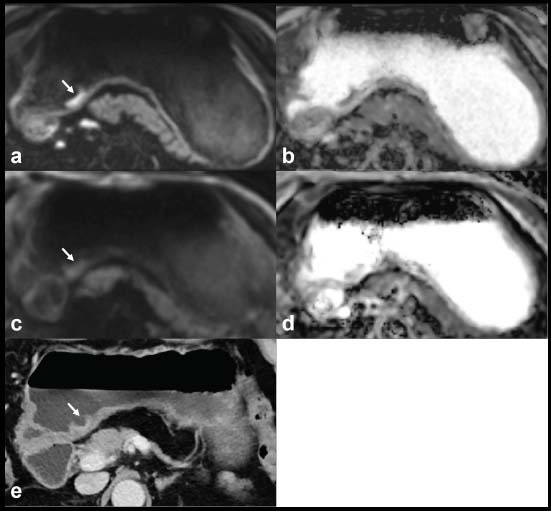
CT and MR images from a 71-year-old male patient with pathologically confirmed adenocarcinoma (stage pT1bN0M0) of the gastric antrum. The EGC tumor is clearly visible as a hyperintense region on the rFOV DWI (**a**, white arrow) and demonstrates hypointensity on the corresponding ADC map (**b**). Conversely, on the fFOV DWI, the tumor appears slightly hyperintense (**c**, white arrow), with hypointensity observed on the corresponding ADC map (**d**). CT imaging reveals focal enhancement in the inner layer of the gastric wall (**e**).

**Fig. (3):**
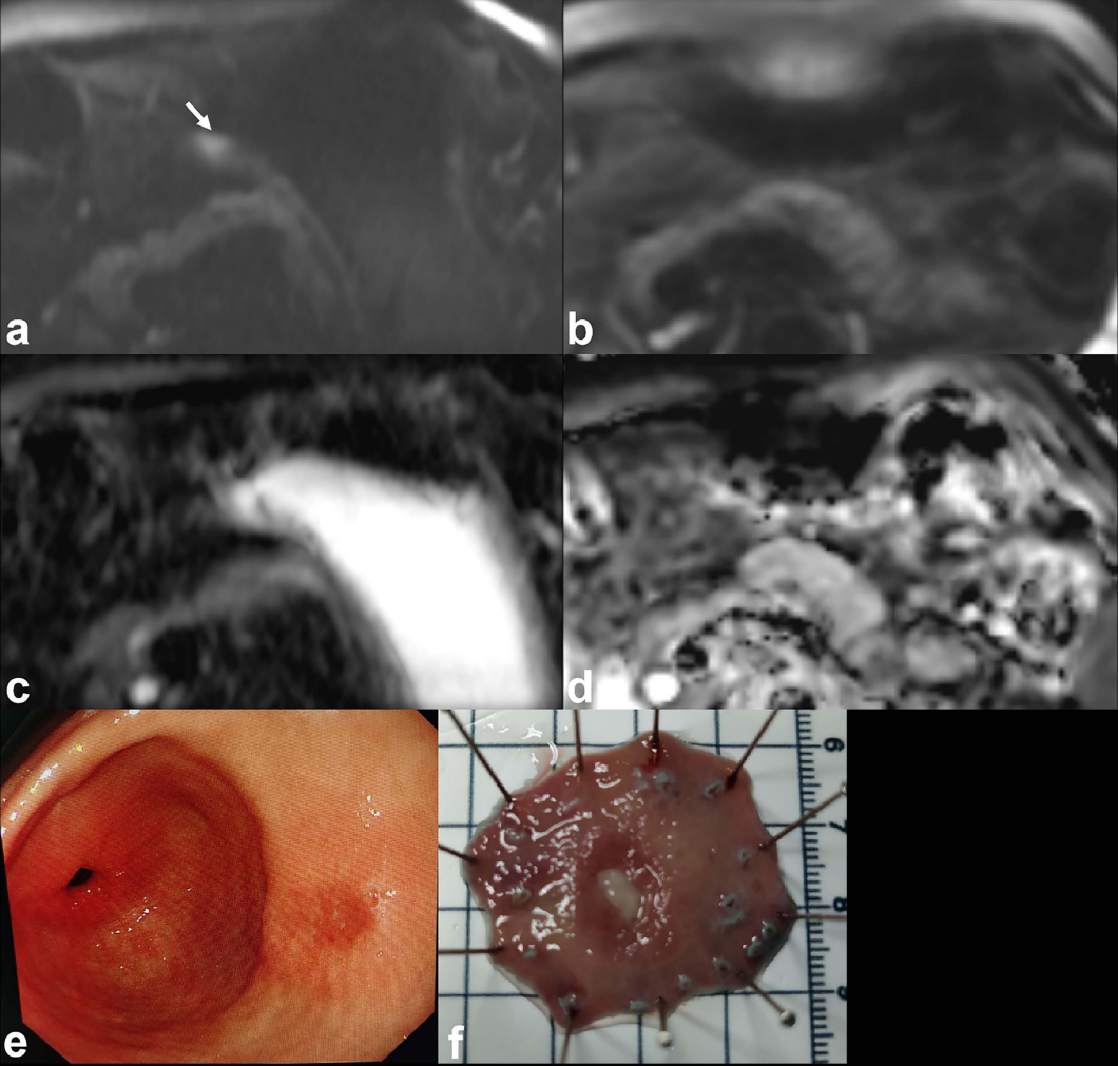
MR images from a 72-year-old female patient with pathologically-confirmed adenocarcinoma (stage pT1aN0M0) of the gastric antrum. The EGC tumor is detected on the rFOV DWI (**a**, white arrow), but remains undetectable on both the fFOV DWI image (**b**) and its corresponding ADC map (**d**). The ADC map corresponding to the rFOV DWI shows isointensity (**c**). Endoscopic examination reveals early gastric cancer on the posterior wall of the gastric antrum (**e**), and the ESD specimen reveals an II-C type lesion (**f**).

**Fig. (4):**
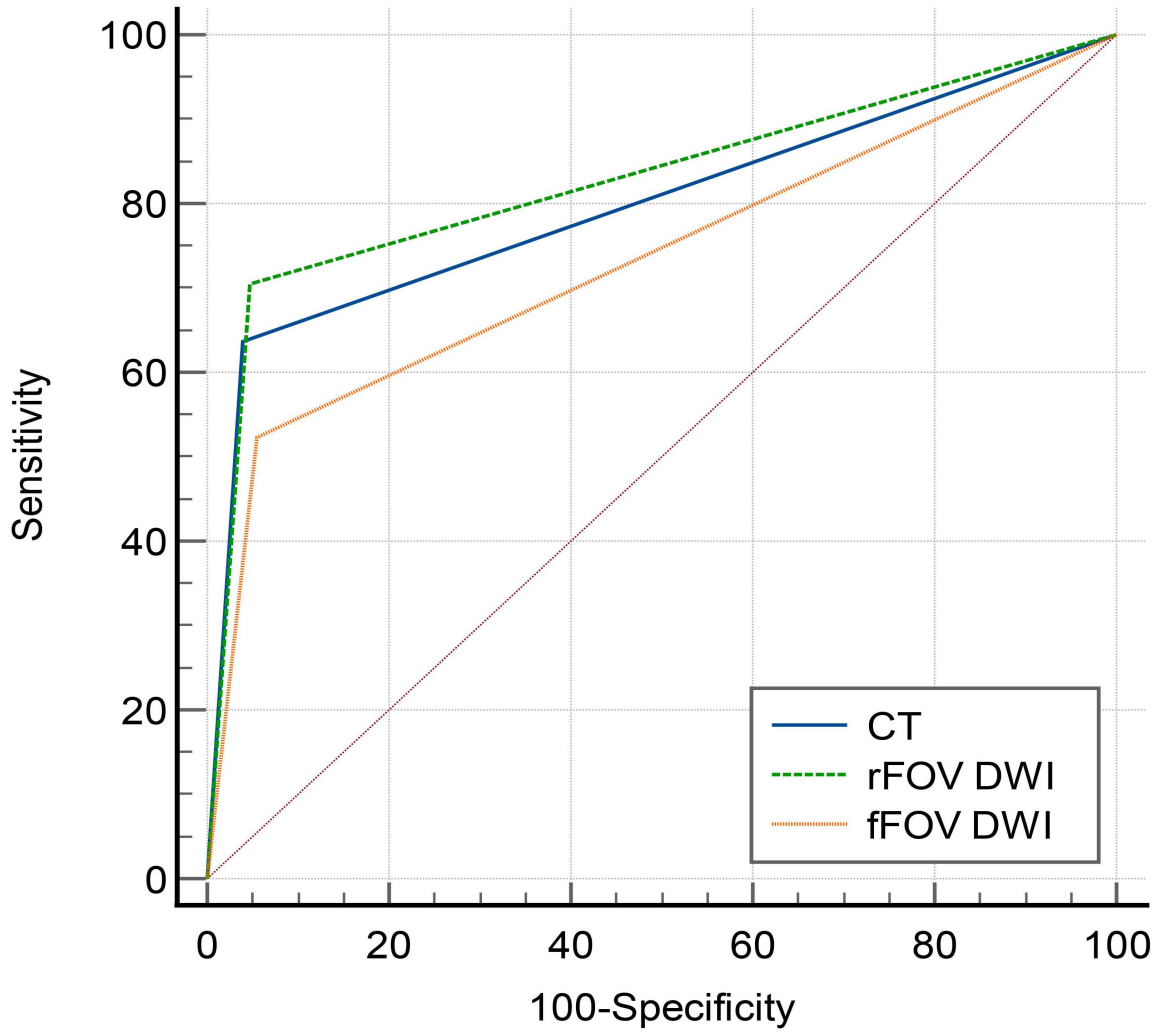
Graph showing ROC curves obtained by rFOV DWI (AUC, 0.829), fFOV DWI(AUC, 0.734), and CT (AUC, 0.799). Better diagnostic performance is achieved using rFOV DWI in EGC patients.

**Table 1:** Characteristics of participants included in this study (n).

**Variable**	**Value**
Mean age (y)*	60.02 ± 9.56
Sex	-
Male	26
Female	17
Body mass index (BMI)*	24.05 ± 3.62
Tumor location	-
Cardia or fundus	12
Body	6
Antrum	24
Pylorus	2
Histopathology	-
Adenocarcinoma	41
Signet-ring cell carcinoma	3
Pathologic T stage	-
pT1a	19
pT1b	25
Tumor size of pathological gross specimen (cm)*	1.87 ± 0.98
Operation	-
Endoscopic resection	4
Radical gastrectomy	39

**Note:** *Data are means ± SDs.

**Table 2:** Comparisons of qualitative image parameters between rFOV and fFOV DWI sequences (Mean ± SD).

**Image parameter**	**rFOV DWI**	**fFOV DWI**	***p*-value**
SNR	57.07±17.62	39.93±16.58	0.021
CNR	12.02±3.24	7.80±3.32	0.004
Overall image quality*	3.26±0.56	2.89±0.43	0.035
Lesion conspicuity*	3.32±0.80	2.18±0.85	<0.001
Anatomic detail*	3.58±0.45	2.39±0.54	<0.001
Artifacts*	3.24±0.51	2.61±0.46	0.004
Lymph node detection*	3.61±0.49	3.34±0.58	0.174
ADC values(×10^-3^mm^2^/s)	1.16±0.19	1.33±0.21	<0.001

Effect sizes (Cohen’s d) for SNR and CNR differences: SNR (d=1.00), CNR (d=1.29). SNR, Signal-to-noise ratio; CNR, contrast-to-noise ratio; rFOV, reduced field-of-view; fFOV, full field-of-view; ADC, apparent diffusion coefficient; DWI, diffusion-weighted imaging. *:1= poor, 2=fair, 3=good, 4=excellent.

**Table 3:** The diagnostic performance of rFOV DWI, fFOV DWI, and CT for EGCs.

	**AUC**	**Sensitivity**	**Specificity**	**Accuracy**
rFOV DWI	0.829(0.764, 0.882)	0.705(0.548, 0.832)	0.953(0.901, 0.983)	0.890
fFOV DWI	0.734(0.661, 0.798)	0.523(0.367, 0.675)	0.945(0.891, 0.978)	0.837
CT	0.799(0.731, 0.856)	0.636(0.478, 0.776)	0.961(0.911, 0.987)	0.878

Data were presented as value (95% confidential interval). rFOV, reduced field-of-view; fFOV, full field-of-view; AUC, area under the curve; CI, confidence interval; DWI, diffusion-weighted imaging.

**Table 4:** Comparisons of ADC values between rFOV and fFOV DWI sequences (Mean ± SD, ×10^-3^mm^2^/s).

**Image parameter**	**T1a stage**	**T1b stage**	***p*-value**
rFOV	1.16±0.21	1.21±0.20	0.506
fFOV	1.64±0.47	1.32±0.21	0.201

rFOV, reduced field-of-view; fFOV, full field-of-view; ADC, apparent diffusion coefficient; DWI, diffusion-weighted imaging.

## Data Availability

The data and supportive information are available within the article.

## LIST OF ABBREVIATIONS

ADC: Apparent Diffusion Coefficient
AJCC: American Joint Committee on Cancer
AUC: Area Under the Curve
CNR: Contrast-to-Noise Ratio
DWI: Diffusion-Weighted Imaging
EGC: Early Gastric Cancer
EPI: Echo-Planar Imaging
fFOV: Full Field-Of-View
MDCT: Multidetector Computed Tomography
MRI: Magnetic Resonance Imaging
rFOV: Reduced Field-Of-View
ROC: Receiver Operating Characteristic
ROI: Region of Interest
SNR: Signal-to-Noise Ratio

## STANDARDS OF REPORTING

STROBE guidelines were followed.
