# Supplementary material for: Reduced Field-of-view Diffusion-Weighted Magnetic Resonance Imaging for Detecting Early Gastric Cancer: A Pilot Study Comparing Diagnostic Performance with MDCT and fFOV DWI
Source: Curr Med Imaging. 2025 Sep 24;21:e15734056390767. doi: 10.2174/0115734056390767250917221319 (PMC13126301; doi:10.2174/0115734056390767250917221319)
Supplement: Supplementary file 1 [file CMIM-21-E15734056390767_SD1.pdf]

1573-4056/25 *Send Orders for Reprints to reprints@benthamscience.net*

BENTHAM SCIENCE

CrossMark

Current Medical Imaging

Content list available at: https://benthamscience.com/journals/cmir

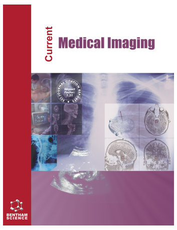

Supplementary Material

Reduced Field-of-view Diffusion-Weighted Magnetic Resonance Imaging for Detecting Early Gastric Cancer: A Pilot Study Comparing Diagnostic Performance with MDCT and fFOV DWI

Guodong Song[1], Guangbin Wang[1], Leping Li[2], Liang Shang[2], Shuai Duan[1], Zhenzhen Wang[3,4] and Yubo Liu[1,*]

[1]*Department of Radiology, Shandong Provincial Hospital Affiliated to Shandong First Medical University, Jinan, Shandong, China*
[2]*Department of Gastrointestinal Surgery, Shandong Provincial Hospital Affiliated to Shandong First Medical University, Jinan, Shandong, China*
[3]*Department of Radiology, People's hospital of Rongcheng, Rongcheng City, Shandong, China*
[4]*Department of Radiology, Shandong Provincial Hospital, Cheeloo College of Medicine, Shandong University, Jinan, Shandong, China*

Table S1. Characteristics of missed lesions on rFOV DWI and fFOV DWI

Characteristic	rFOV DWI-Missed Lesions (n=13)	fFOV DWI-Missed Lesions (n=21)	rFOV DWI-Detected Lesions (n=31)
**Size (cm)***	1.24 ± 0.57	1.51 ± 0.78	2.13 ± 0.98
**Location, n (%)**	-	-	-
- Antrum	9 (69.2)	11 (52.4)	15 (48.4)
- Body	2 (15.4)	4 (19.0)	4 (12.9)
- Cardia/Fundus	2 (15.4)	5 (23.8)	10 (32.3)
- pylorus	0	1 (4.8)	2 (6.5)
**Pathologic Stage, n (%)**	-	-	-
- T1a	8 (61.5)	11 (52.4)	11 (35.5)
- T1b	5 (38.5)	10 (47.6)	20 (64.5)

* mean ± SD. Lesion size differed significantly between rFOV DWI-missed and detected groups (*t*-test, $P = 0.004$).

© 2025 The Author(s). Published by Bentham Science Publisher.

This is an open access article distributed under the terms of the Creative Commons Attribution 4.0 International Public License (CC-BY 4.0), a copy of which is available at: https://creativecommons.org/licenses/by/4.0/legalcode. This license permits unrestricted use, distribution, and reproduction in any medium, provided the original author and source are credited.

DOI: 10.2174/0115734056390767250917221319, 2025, *21*, e15734056390767
